# Investigating the relationship between interleukin‐6 serum levels and outcome in acute ischemic CVA

**DOI:** 10.1002/brb3.1668

**Published:** 2020-06-25

**Authors:** Arash Mosarrezaii, Mohammad Reza Amiri‐Nikpour, Hamid Reza Mehryar, Babak Choobi Anzali, Sarmad Nourooz‐Zadeh, Shadi Babaei, Hedieh Farrokhi

**Affiliations:** ^1^ Department of Neurology Urmia University of Medical Sciences Urmia Iran; ^2^ Clinical Research Development Unit of Imam Khomeini Hospital Urmia University of Medical Sciences Urmia Iran; ^3^ Department of Emergency Medicine Urmia University of Medical Sciences Urmia Iran; ^4^ Urmia University of Medical Sciences Urmia Iran

**Keywords:** inflammatory factors, interleukin‐6, ischemic stroke, serum level, stroke severity

## Abstract

**Introduction:**

Interleukin‐6 (IL‐6) is among the inflammatory mediators exhibiting elevated levels in ischemic stroke (IS) patients. The present study set out to evaluate the relationship between serum levels of interleukin‐6 with long‐term and at‐hospital outcomes of acute ischemic stroke in patients hospitalized at Imam Khomeini Hospital, Urmia, Iran, from 2017 to 2018.

**Method and materials:**

This cross‐sectional descriptive study enrolled 29 and 31 acute stroke patients for long‐term and at‐hospital observation, respectively. Evaluation of stroke severity was performed using the National Institute of Health Stroke Scale (NIHSS) and the modified Rankin Scale (mRS) on days 1, 5, and 90. Serum IL‐6 level was measured via enzyme‐linked immunosorbent assay (ELISA) on days one and five.

**Results:**

In the present cohort study, the following population were enrolled: for long‐term evaluation, 11 (38%) men and 18 (63%) women with a mean age of 64.5 ± 14.9 years, and for at‐hospital evaluation: 11 (37.5%) men and 20 (64.5%) with a mean age of 65.25 ± 14.37 years. A significant positive correlation was observed between IL‐6 levels with NIHSS and mRS scores of the patients from time of admission until the end of the follow‐up period (long‐term: *p* < .001; at‐hospital: 0.022).

**Conclusion:**

The evidence from the present study suggests that IL‐6 contributes to the determination of the severity of ischemic strokes and may be useful in predicting prognosis. However, larger scale studies are required to further establish these finds.

## Graphical abstract

It shows that IL‐6 contributes to determination of severity of ischemic stroke and may be useful in predicting patient prognosis.

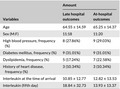

## 1 INTRODUCTION

A stroke is generally referred to a syndrome of acute neuronal defect lasting for at least 24 hr, resulting from cerebrovascular disruption and ultimately leading to focal involvement within the central nervous system (Barnett, 2005). Following cancer and coronary artery diseases, cerebrovascular accidents are the third global leading cause of death claiming approximately 700,000 annual victims within the United States (600,000 ischemic and 100,000 hemorrhagic; overall mortality rate: 12%) (Baldwin et al., 2010). According to a survey conducted in the United States, more than 20% of patients admitted to the neurology department of general hospitals suffer from a variation of coronary artery disease (Ropper, 2005). In Iran, annual stroke incidence is 327:100,000, also claiming first place among leading causes of disability in the country. According to the Public Relations Department of the Ministry of Health and Medical Education of Iran, cardiovascular diseases account for 39.3% of the country's deaths, 9.3% of which result from strokes (Mirzaei et al., 2012). Eighty‐eight per cent of stroke cases are ischemic and 8%–12% of these strokes lead to death within a month. Many inflammatory responses are mediated by cytokines and small glycoproteins expressed by an array of cells in response to acute cerebral ischemia (Bharosay, Saxena, Varma, Bharosay, & Pandey, 2011). Recent studies have established that, in addition to their ability to produce cytokines and chemokines, brain cells possess the ability to express cytokine receptors capable of triggering intrathecal inflammatory reactions (Barone, 1999). Interleukin‐6 (IL‐6) is an inflammatory cytokine involved in the pathogenesis of various neurological disorders, including strokes (Suzuki & Tanaka, 2009), acting as a messenger molecule between leukocytes, vascular endothelium, and parenchymal cells (Dziedzic, Slowik, & Szczudlik, 2003; Gertz et al., 2012). Following secretion from multiple sources including monocytes, macrophages, fibroblasts, endothelial cells, keratinocytes, mast cells, T lymphocytes, and microglia and astrocytes (Cojocaru et al., 2009), IL‐6 is capable of apoptosis inhibition, stimulation of differentiation and inhibition of growth due to the cellular context (Dziedzic et al., 2003; Gertz et al., 2012). The roe of IL‐6 is very important in responses to neuronal damage (Baldwin et al., 2010). According to in vitro investigations, IL‐6 inhibits differentiation of neuronal precursor cells (Monje, Toda, & Palmer, 2003; Nakanishi et al., 2007). It is also influential in the activation of microglia and astrocyte in addition to the regulation of nervous neuropeptide excretion (Baldwin et al., 2010). An array of pro‐inflammatory properties such as induction and promotion of secondary inflammatory responses within the brain tissue and pertaining vessels has also been discovered. Furthermore, it has been revealed that IL‐6 is a key mediator in inflammatory responses to cerebral ischemia (Cojocaru et al., 2009). IL‐6 can be measured as soon as one hour following the occlusion of middle cerebral artery (MCA) in the experimental models of brain ischemia (Yamasaki et al., 1995).

A number of studies have introduced IL‐6 as an early marker of inflammation in the setting of acute ischemic strokes (Shenhar‐Tsarfaty et al., 2008). In several studies, higher levels of CRP and IL‐6 were associated with poor clinical outcomes following both ischemic and hemorrhagic strokes (Castillo et al., 2002; Mirzaei et al., 2012,; Whiteley, Chong, Sengupta, & Sandercock, 2009). Hence, IL‐6 has been proposed as a prognostic marker in acute ischemia of both hemorrhagic and ischemic natures (Waje‐Andreassen et al., 2005). Based on a study conducted by Mehryar, Khoshakhlagh, Shadfar, Khaffafi, and Rezazadeh (2019), a positive clinical history of diabetes mellitus, hypertension, dyslipidemia, myocardial infarction, chronic heart failure, coronary artery bypass, angioplasty/stenting, and smoking was significantly correlated with ischemic stroke‐associated mortality (Mehryar et al., 2019). Oto et al. (2008) performed a study on 37 patients with acute cerebral stroke regarding the relationship between one‐month outcome and serum levels of IL‐6, among a number of other biomarkers. It was reported that serum interleukin‐10 and more significantly six levels were correlated with poor 1‐month outcome (Oto et al., 2008). Meanwhile, some studies, such as that by Suzuki and Tanaka (2009), have reported neuroprotective, antiapoptotic, and regenerative properties for IL‐6 in the setting of cerebral ischemia (Suzuki & Tanaka, 2009).

A study conducted by Fahmi et al. (Egypt; 2016) set out to identify independent predictors of short‐term outcome of the first ischemic stroke in young adults. Precise medical, neurological, and laboratory data were collected, all of which were revealed to be significantly correlated with the NIHSS score. Multiple regression analysis showed that the size of the infarct area (as determined via CT scan) as well as IL‐6 levels and their synergistic effects were important predictors for the NIHSS results. IL‐6 and the size of the ischemic region were revealed to be independent predictors of premature stroke short‐term outcomes following premature stroke in young adults. Also, reducing Ll‐6 levels could possibly limit the damage at the early phases of stroke (Fahmi & Elsaid, 2016). In a study by Feng et al. (2016) investigating the neuroprotective properties of IL‐6 in a rat model of cerebellar ischemic, intracerebral obstruction of the middle cerebral artery was performed. After receiving either 50 or 500 ng of IL‐6, lesion size and neurodegenerative symptoms were evaluated. Studies have shown that IL‐6 inhibits neoplastic apoptosis in in vivo settings. In vitro investigations have also reported that IL‐6 is capable of the phosphorylation of STAT3 (signal transducer and activator of transcription 3) as well as the expression of a protein that responsible in the differentiation of myeloid leukemic cells (MCL1). However, it failed to exhibit any influence on the expression of B‐cell lymphoma 2. Overall, these findings indicate that Janus kinase STAT3 pathway is activated by IL‐6, also playing a role in the regulation of cytokine secretion as well as the permeability of the blood–brain barrier in settings of cerebral ischemia. IL‐6 is capable of reducing the serum levels of an array of inflammatory cytokines including TNF‐α and interleukin‐1β and myeloperoxidase, which results in the accumulation of granulocytes within the ischemic tissue. The results of this study showed that neuroprotective properties of IL‐6 in cerebral ischemia are mainly due to the regulation of apoptosis, cytokine secretion, and the preservation of the integrity of the blood–brain barrier. Hence, IL‐6 has been proposed as a therapeutic agent in the setting of cerebral stroke (Bilic, Dzamonja, Lusic, Matijaca, & Caljkusic, 2009; Oto et al., 2008).

The high mortality and morbidity rates of cerebral stroke impose a significant financial burden on supportive health systems. Coupled with the established short‐term predictive value of IL‐6, this study was designed to evaluate the at‐hospital and long‐term predictive value of serum IL‐6 values obtained on days one and fifth postcerebral stroke (PCS) in a population of Iranian adults admitted to Imam Khomeini Hospital emergency department (Urmia, Iran) following the incidence of an ischemic.

## 2 PROJECT IMPLEMENTATION METHOD

### 2.1 Research method

This study was approved by the ethics committee of Urmia University of Medical Sciences (ethics codes: Irumsu.rec.1395.451 & Irumsu.rec.1395.493). All patients admitted to the emergency department (ED) with a diagnosis of acute cerebral stroke were investigated over a period of one year. The inclusion criteria were as follows:

1. Acute focal ischemic stroke as proven by clinical examination and imaging
2. Acute stroke located in regions corresponding to the anterior circulation of the brain (ACA and MCA and internal carotid)
3. Age >18 years
4. Admission to the E.D. <24 hr from the onset of symptoms
5. Complete examination of neurological performance at the time of admission to the ED
6. Personal consent of the patient.

Exclusion criteria comprised of the following:

1. Hemorrhagic stroke
2. Resolution of symptoms within 24 hr of onset
3. Admission of arterial or venous TPA
4. Previous history or current evidence of other neurological diseases (e.g., brain tumors and demyelination diseases.)
5. Craniotomy, severe brain damage, and trauma
6. Positive history of infectious or inflammatory diseases (e.g., malignancies and vascular collagen diseases)
7. Antibiotics or anti‐inflammatory therapy administered within the last 72 hr
8. NIHSS >20.

A complete neurologic examination was performed on all patients at the time of admission to the ED. In addition, the NIHSS and mRS were also completed for each individual upon arrival.

In addition to on‐site screening, data regarding age, sex, risk factors of atherosclerosis, and nature of ischemia were gathered via checklists. Serum levels of IL‐6 were measured according to the ELISA procedure on days 1 and 5 PCS. Follow‐up was performed 90 days after admission, and the evaluation included NIHSS and mRS index assessment as well as evaluation of motility and neurological function. Following data collection, the relationship between serum IL‐6 level and the late and at‐hospital outcomes of acute focal ischemic stroke was analyzed by statistical analysis.

### 2.2 Statistical analysis

Evaluation was performed using SPSS software v.20 (IBM). In order to assess the distribution of the data, the Kolmogorov–Smirnov normality test was employed. Normally and otherwise distributed data were analyzed using respective tests. Quantitative data were expressed as mean ± *SD* or median, while qualitative data were expressed as percentages and frequencies. *p*‐values <.05 were determined to be statistically significant.

### 2.3 Research type

Cohort.

### 2.4 Research population

Hospitalized patients diagnosed with acute focal ischemic stroke in Imam Khomeini Hospital, Urmia, Iran, between 2017 and 2018.

### 2.5 Ethical approval

This study was approved by the Medical Ethics Committee of Urmia University of Medical Sciences (Irumsu.rec.1395.451 and Irumsu.rec.1395.493).

## 3 RESULTS

Demographic information of the studied patients is presented in Table 1.

**TABLE 1:** Demographic information of the studied patients

Variables	Amount	
	Late hospital outcomes	At‐hospital outcomes
Age	64.55 ± 14.59	65.25 ± 14.37
Sex (M:F)	11:18	11:20
High blood pressure, frequency (%)	8 (27.86%)	9 (29.03%)
Diabetes mellitus, frequency (%)	9 (31.01%)	9 (31.01%)
Dyslipidemia, frequency (%)	5 (17.24%)	7 (22.58%)
History of heart disease, frequency (%)	3 (10.34%)	3 (10.34%)
Interleukin at the time of arrival	10.85 ± 12.77	12.82 ± 13.53
Interleukin (fifth day)	18.84 ± 32.73	13.93 ± 13.37

**FIGURE 1:**
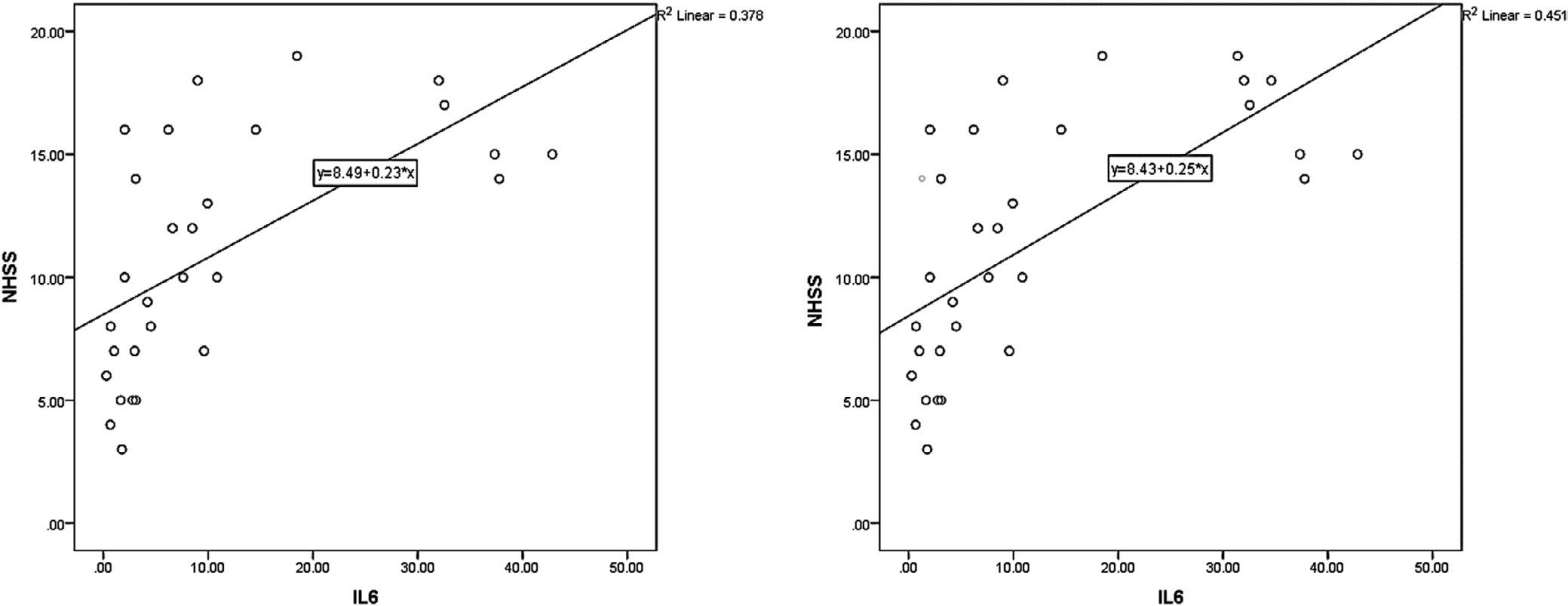
Correlation between first day IL‐6 and NHSS

According to the results, the mean NIHSS value on the first days PCS was 11.00 ± 4.81 and 11.43 ± 5.01 for late and at‐hospital outcomes, respectively (Figure 1). The relationship between IL‐6 levels and NIHSS was evaluated on the first day PCS, revealed to hold statistical significance (*p* = .004, *r* = .381 and *p* < .001, *r* = .672 for late and at‐hospital outcomes). Patients with higher IL‐6 exhibited higher NIHSS.

**FIGURE 2:**
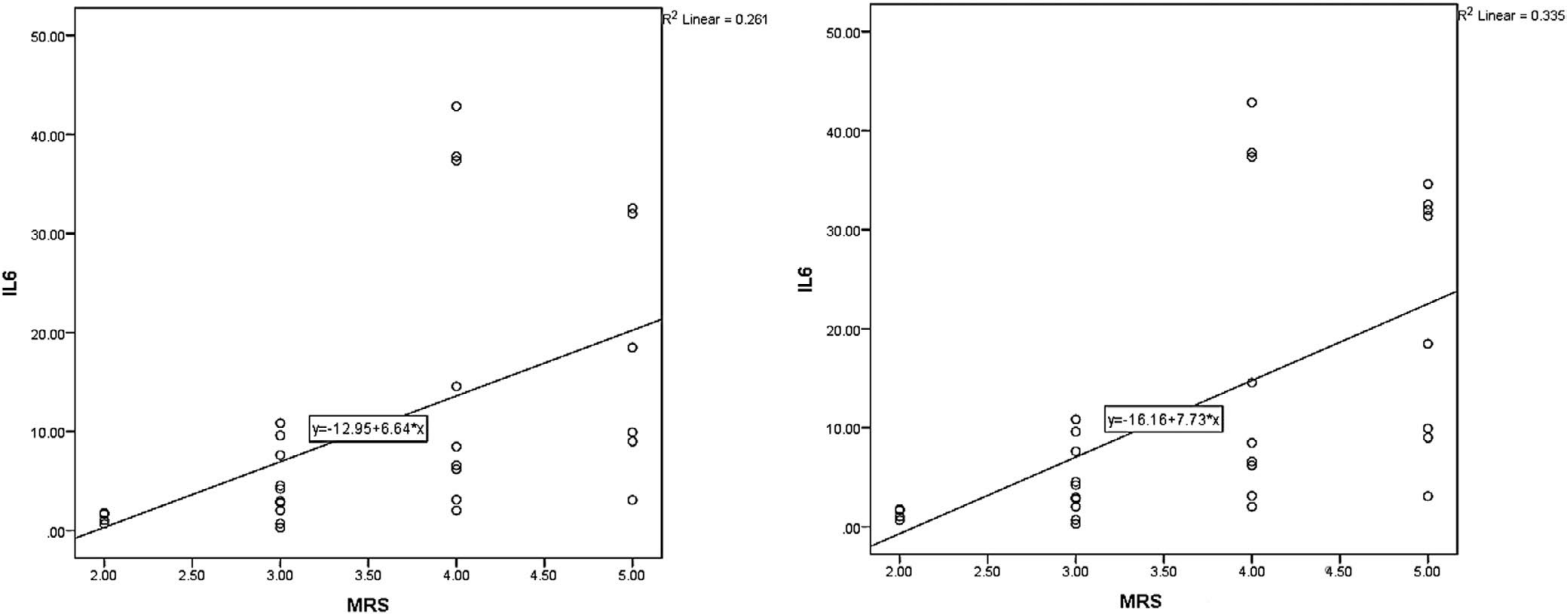
The relationship between mRS and IL‐6 in the first day

In this study, the mean modified Rankin Scale (mRS) value in the first day PCS was 3.58 ± 0.98 and 3.67 ± 1.01 for long‐term and at‐hospital outcomes, respectively. The correlation between mRS values at day 1 PCS with IL‐6 values was deemed statistically significant (*p* = .005, *r* = .511, and *p* = .005, *r* = .510, for long‐term and at‐hospital outcomes). Figure 2, shows the relationship between mRS and IL‐6 on day one PCS.

**FIGURE 3:**
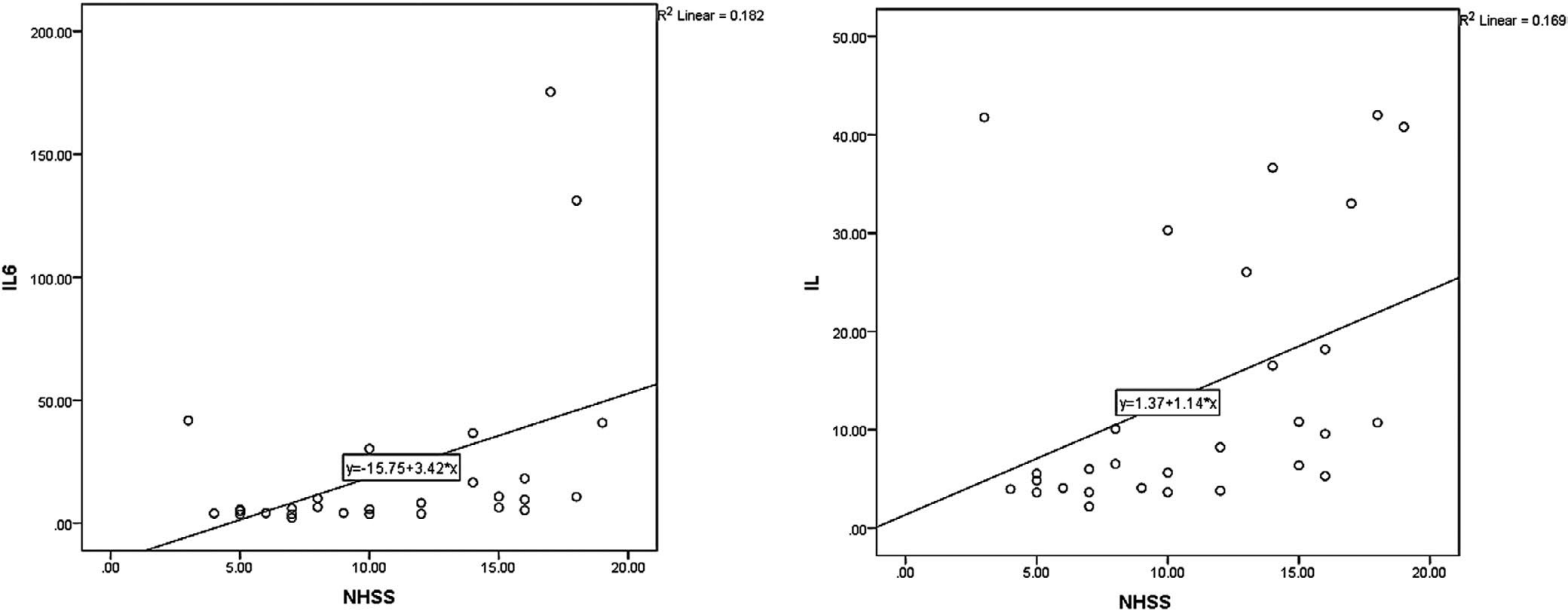
The correlation between IL‐6 (in the 5th day) and NIHSS (in the 1st day)

At day seven, the mean serum IL‐6 values were 18.84 ± 32.73 and 13.93 ± 13.37 for long‐term and at‐hospital outcomes, respectively, exhibiting a statistically significant correlation with NIHSS values on day one PSC (*p* = .040, *r* = .362, and *p* = .021, *r* = .411, for late and at‐hospital outcomes; Figure 3).

**FIGURE 4:**
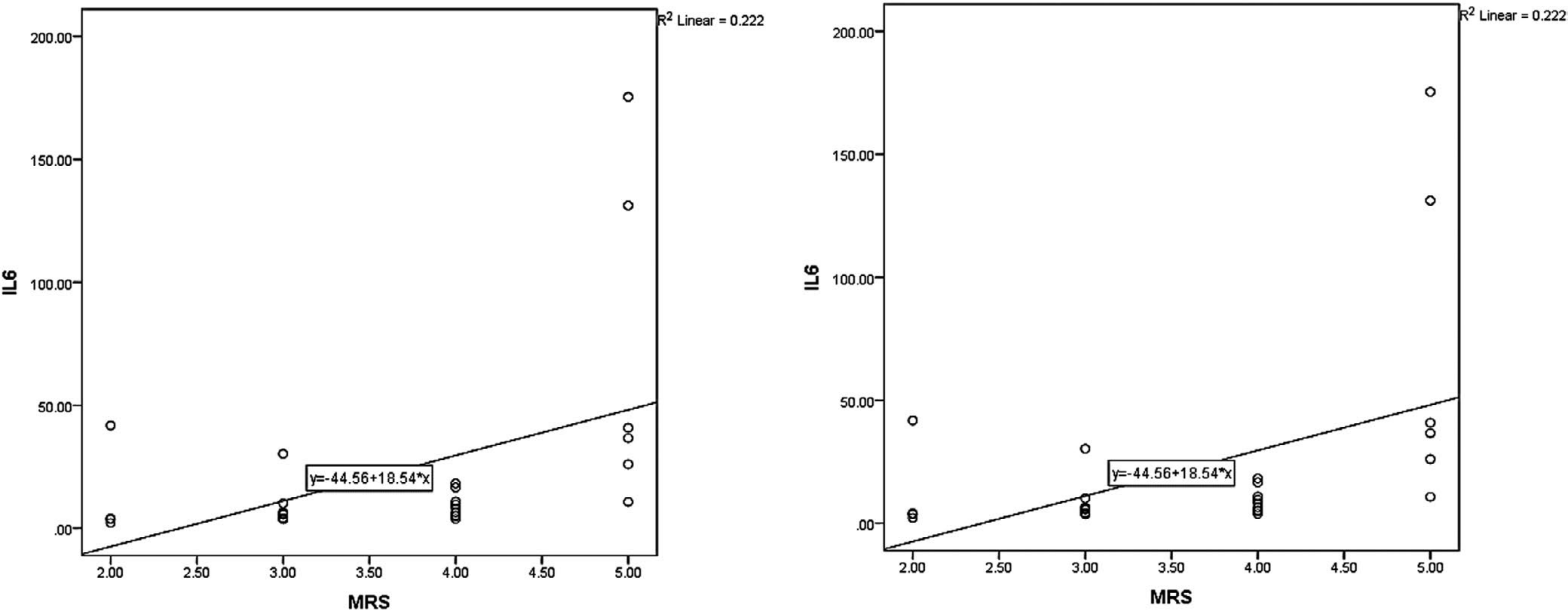
The relationship between IL‐6 in the fifth day and mRS in the first day

It was found that IL‐6 values on day seven PCS were directly correlated with mRS values obtained on day one PCS (*p* = .025, *r* = .416 for both late and at‐hospital outcomes; Figure 4).

Regretfully, a patient met their demise throughout the course of the study. Since long‐term follow‐up and NHISS and mRS assessment was not possible, the data gathered from this patient were excluded from the study. However, IL‐6 values at day one and five PCS were considerably high in the deceased patient (32.54 and 40.62, respectively). The mean NHISS value 90 days PCS was 6.78 ± 4.85, exhibiting direct correlation with baseline IL‐6 values (*p* < .001, *r* = .679).

**FIGURE 5:**
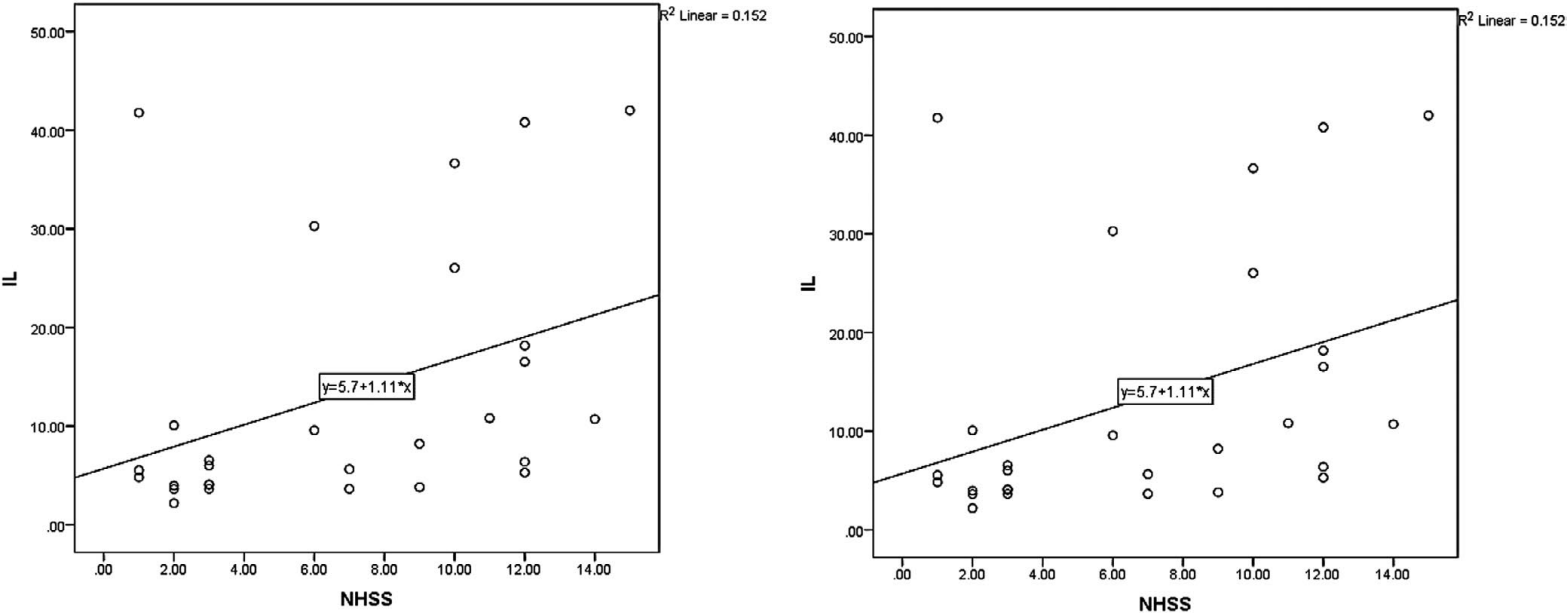
The correlation between IL‐6 in the first day with NHISS (at the third month and fifth day for late and intrahospital outcomes, respectively)

In case of at‐hospital outcomes, the mean NHISS score in patients on the fifth day was 8.65 ± 5.03 that showed significant correlation with IL‐6 in the first day (*p* < .001 and *r* = .654). Figure 5 depicts the evolution of IL‐6 relative to NHISS.

**FIGURE 6:**
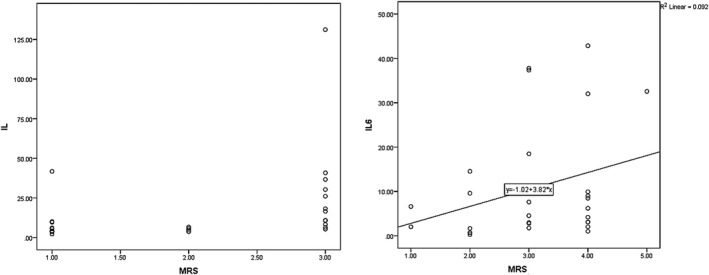
The correlation between IL‐6 in the first day and mRS in the third month and fifth day for late and intrahospital outcomes, respectively

According to Figure 6, mean mRS values for long‐term and at‐hospital outcomes were 2.03 ± 0.92 and 3.10 ± 1.01, respectively. The relationship between mRS at 90 days PCS and day 5 PCS indicated a significant direct correlation between the two (*p* = .01, *r* = .613, and *p* = .101, *r* = .303, for late and intrahospital outcomes; Figure 6).

**FIGURE 7:**
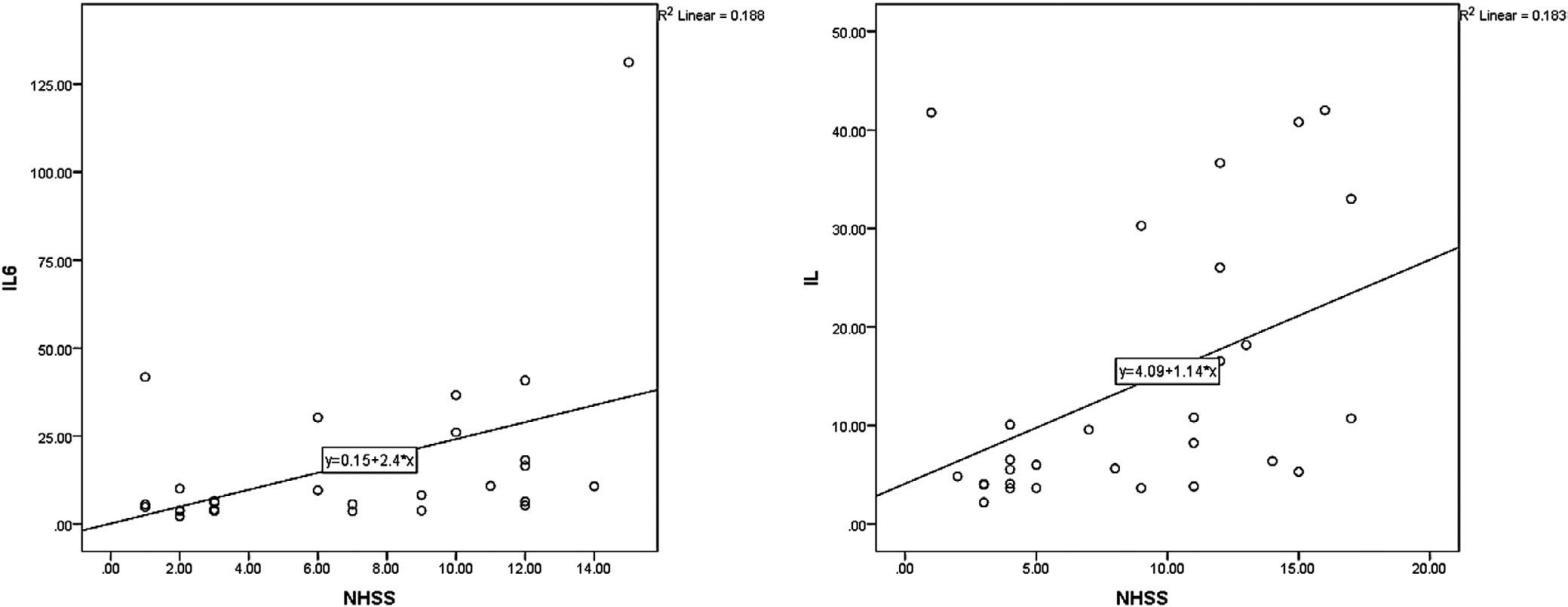
The correlation between IL‐6 in the 5th day and NIHSS in the third month and 5th day for late and intrahospital outcomes, respectively

**FIGURE 8:**
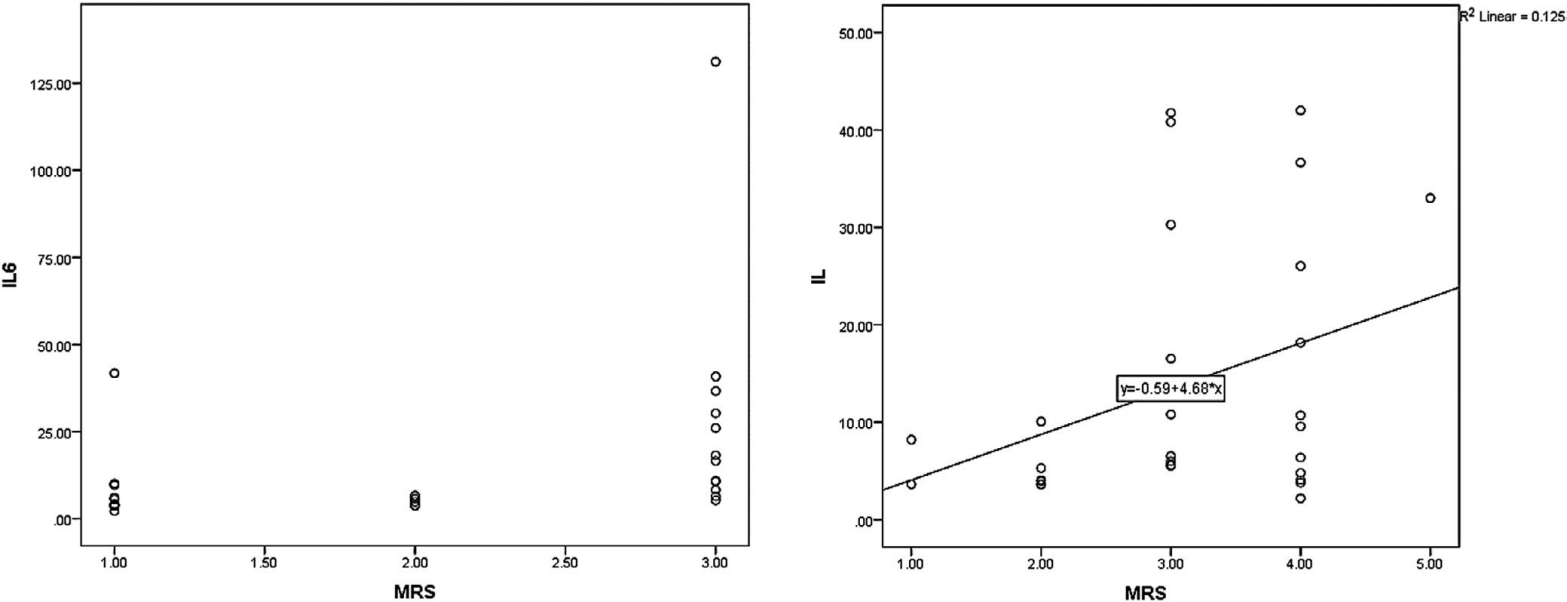
The correlation between IL‐6 in the 5th day and mRS in the third month and 5th day for late and intrahospital outcomes, respectively

Analysis revealed the relationship between the IL‐6 value on day 5 PCS with NHISS at day 90 PCS to be significant (*p* = .040, *r* = .390, and *p* = .021, *r* = .428, for long‐term and at‐hospital outcomes). Also, the relationship between IL‐6 on day 5 and mRS was statistically significant for late hospital outcomes (*p* = .019, *r* = .442). The same failed to apply for at‐hospital outcomes (*p* = .059, *r* = .354). Figures 7 and 8 depict the correlations between IL‐6 with neurological criteria of NHSS and mRS.

In intrahospital outcomes, two patients met their demise. Mean IL‐6 values at baseline were reported to be 33.01 ± 2.26 in the deceased and 10.85 ± 12.77 in remainder of the patients. The values for the former group were significantly higher than the latter (*p* = .022).

## 4 DISCUSSION

A body of evidence suggests that ischemic stroke results in inflammation, leading to an increase in C‐reactive protein (CRP) and various cytokines serum levels. It has been proposed that serum levels of IL‐6 in the first 24 hr are directly related with the improvement of the neurological symptoms. On the other hand, however, some studies have provided opposing evidence (Bilic et al., 2009; Bustamante, Simats, Vilar‐Bergua, García‐Berrocoso, & Montaner, 2016; Cojocaru et al., 2009; Esenwa & Elkind, 2016; Lakhan, Kirchgessner, & Hofer, 2009). Therefore, in the present study, we decided to investigate the relationship between serum IL‐6 level at days one and seven PCS with the long‐term outcome of acute ischemic stroke. Empirical studies indicate that in acute stroke, inflammatory mediators (such as interleukin‐1 and IL‐6 and tumor necrosis) are released within 2 hr from the onset of insult as a direct response to brain damage. In addition, anti‐inflammatory therapies have yielded positive results in the treatment of affected patients (Beamer, Coull, Clark, Hazel, & Silberger, 1995). In a study by Beamer et al. (1995), following the omission of results obtained from infected patients, an increase was observed in both IL‐6 and CRP values in infarcted patients, as evidenced by CT scan. The same results were not recreated in patients with lacunar stroke. A significant direct correlation was also observed between increased levels of IL‐6 and NHISS and mRS values obtained at baseline, which is most likely to proceed evidence of infarction obtained through CT scan at ultimately 12 hr after admission to the emergency department. The evidence suggests that the results of the study by Beamer et al. (1995) show the association between IL‐6, CRP, and cerebral infarction.

In general, inflammation is associated with increases in CRP and IL‐6. A further possible cause could be plaque atherosclerosis. The role of viral diseases in atherogenesis in ischemic heart disease has been established; however, this influence remains to be vague in carotid artery diseases. Inflammatory cells, especially macrophages, cause the synthesis of cytokines before local and systemic inflammation following an infectious of arterial infection with the rupture of atherosclerotic plaques similar to coronary artery disease (Beamer et al., 1995).

Cardiac embolism, another known mechanism of acute cerebral stroke, can be commonly found in stroke patients prior to onset of CVA (Cerebrovascular Accident). In the majority of cardiac ischemic diseases, fetal inflammatory proteins are elevated, indicating that interleukins could possibly be indicative of instability of atherosclerotic plaques (Muir, Weir, Alwan, Squire, & Lees, 1999).

Systemic infections are common in stroke patients with many patients exhibiting signs of infection in the weeks prior to the occurrence of CVA. Acute phase proteins, especially IL‐6 and fibrinogen, are potent thrombotic stimulants which may result in acute thrombosis at the site of previously available atherosclerosclerotic plaques. CRP and IL‐6 values are associated with *Chlamydia pneumoniae* and *Helicobacter pylori* antibody titers in the general population as well as increased risk of cardiovascular accidents. The increased levels of IL‐6 in the setting of MI combined with the rapid progression of cerebral and cardiac events in patients with increased IL‐6 support this evidence (Di Napoli, Papa, & Bocola, 2001; Vila, Castillo, Dávalos, & Chamorro, 2000).

A significant number of studies have been published on the role of inflammatory markers in stroke. Molecular markers of inflammation are useful in managing patients with ischemic stroke during the acute phase as well as improving prognosis and risk prevention (Meng, Zhang, Shi, Zhang, & Yuan, 2015). To clarify, inflammatory cytokines, such as IL‐6 and tumor necrosis factor alpha (TNF‐α), result in an increased infarct size and the premature development of neurological symptoms.

In a study that evaluated patients after acute ischemic stroke at days one and 8 PCS, it was reported that IL‐6 not only predicts both stroke and infarct severity and patient prognosis (Rodríguez‐Yáñez & Castillo, 2008). Data analysis revealed that the mean IL‐6 values were significantly higher in patients who had died following the stroke.

The pro‐inflammatory cytokines (IL‐1β, IL‐6, and TNF‐α) are released into the bloodstream following the obstruction of vascular flow in the ischemic area distal to the location of insult, although this process may also play a major role in rebuilding neurons.

Some cytokines, such as IL10, are capable of inhibiting neuronal regeneration and reconstruction processes. A number of studies have shown that low levels of interleukin‐10 are inversely associated with insult severity (Becker, 1998; Rodríguez‐Yáñez & Castillo, 2008; Vila et al., 1999; Woiciechowsky, Schöning, Lanksch, & Volk, 1999). As previously mentioned, IL‐6 was significantly higher in deceased patients, opposing results obtained from other studies. In a study conducted by Oto et al. (2008), researchers failed to report a significant correlation between IL‐6 levels and patient prognosis (Oto et al., 2008). The discrepancies regarding the results of our study and that by Oto el al. may lie in the more limited area of infarction in the latter study. In general, low IL‐6 levels may be indicative of a rather small infarcted region or the absence of brain damage altogether.

Oto et al. (2008) showed that increased levels of IL‐10 and 6 were associated with poorer prognosis in patients with hemorrhagic stroke which was evaluated over the course of a month during this study. Another study showed that interleukin levels are directly related with the volume of the present hematoma. The precise mechanism behind the release of interleukins in hemorrhagic strokes remains unclear. As shown on an animal model of brain damage, IL‐6 and IL‐10 release results from sympathetic activation as well as catecholamine release due to increased intracranial pressure following the occurrence of stroke (Fassbender et al., 1994; Oto et al., 2008).

## 5 CONCLUSION

This study reports a direct correlation between high IL‐6 levels and stroke‐induced damage based on NHSS and mRS criteria by measuring the interleukins of the 1st and 5th days and its association with neurologic criteria on days 1 and 90, and 1 and 5 of evidence for late and intrahospital outcomes, respectively. A number of hypothesis, such as the role of infectious agents in the rupture of atherosclerosis plaques and bacterial causes, have been suggested, and it is hoped that further studies in this regard could help better identify patients with increased risk of stroke. Intrahospital results revealed that IL‐6 level on day one PCS was significantly higher in patients who died prior to evaluation on day 5 PCS compared to survivors, indicating correlation between higher serum IL‐6 values and poorer prognosis in stroke patients.

## CONFLICT OF INTEREST

There is no conflict of interest between authors, research centers, institutions, and any organization.

## AUTHOR'S CONTRIBUTION

All of the authors contributed equally.

## Data Availability

The gathered data will be made available upon request and on the condition of accurate citation.
